# Reactive lymphoid hyperplasia of the eustachian tube orifice presenting with aural fullness: a case report

**DOI:** 10.3389/fsurg.2026.1859585

**Published:** 2026-06-25

**Authors:** Lingping Ding, Qi Wu, Zhaoli Meng

**Affiliations:** Department of Otolaryngology Head and Neck Surgery, West China Hospital, Sichuan University, Chengdu, China

**Keywords:** aural fullness, eustachian tube dysfunction, eustachian tube orifice, lymphoid hyperplasia, skull base mass

## Abstract

Reactive lymphoid hyperplasia (RLH) is a common benign lesion, yet its occurrence at the eustachian tube orifice-a rare skull base site-is poorly reported. Lesions in this anatomical region are predominantly malignant, predisposing clinicians to misdiagnose benign lesions as nasopharyngeal carcinoma and lead to overexamination and therapy. We herein report a 58-year-old male patient presenting with persistent aural fullness and hearing loss caused by a lymphoproliferative mass at the right eustachian tube orifice. A clinical timeline is provided to clarify the disease course. The clinical characteristics, imaging features, endoscopic findings, surgical management, and pathological diagnosis are discussed. This case provides practical insights for the diagnosis and treatment of similar rare lesions, helping to reduce misdiagnosis and overtreatment.

## Introduction

The eustachian tube connects the nasopharynx and middle ear cavity, and its core functions include pressure balance, secretion clearance, and protection against pathogenic invasion (1). Obstruction at the eustachian tube orifice frequently causes eustachian tube dysfunction and otitis media with effusion, manifesting as aural fullness, hearing loss, otalgia, and tinnitus (2, 3).

Most neoplastic lesions arising at the eustachian tube orifice are malignant, such as nasopharyngeal carcinoma and extranodal NK/T-cell lymphoma (4). Benign lymphoproliferative lesions in this region are rare and easily misdiagnosed due to overlapping clinical and imaging features with malignant tumors.

RLH is a benign immune-mediated response to chronic inflammation or stimulation (5). Definitive diagnosis relies on histopathology and immunohistochemistry (6, 7). We present a case of benign lymphoid hyperplasia at the eustachian tube orifice manifesting as isolated aural fullness, emphasizing the importance of comprehensive evaluation and pathological confirmation in clinical practice. This report adheres to the CARE guidelines for case reporting.

## Case presentation

A 58-year-old male patient presented in April 2024 with a 5 month history persistent right-sided aural fullness, accompanied by right hearing loss and occasional tinnitus. He also reported nasal obstruction, yellowish purulent rhinorrhea, nasal itching, and sneezing. He denied otalgia, otorrhea, dizziness, headache, or significant weight change. The patient had no history of diabetes, hypertension, or coronary heart disease, and denied smoking or alcohol abuse. Otoscopy revealed dark tympanic membrane, and middle ear effusion was aspirated; tympanometry was not performed at that visit. He was diagnosed with otitis media with effusion and received symptomatic treatment, but symptoms persisted. In August 2024, repeated nasal endoscopy identified a neoplastic lesion at the right Eustachian tube orifice, and biopsy suggested lymphoid hyperplasia. Due to progressive symptoms, he was referred to our hospital (Table 1).

**Table 1:** Clinical timeline of the patient.

Time	Event
April 2024	Onset of right aural fullness and hearing loss; local hospital diagnosis of otitis media with effusion
August 2024	Nasal endoscopy: right Eustachian tube orifice mass; biopsy: lymphoid hyperplasia
September 2024	Persistent symptoms; referred to our hospital
September 29, 2024	Nasopharyngeal MRI: 1.7 cm enhancing nodule at right Eustachian tube orifice
October 18, 2024	Admission
October 21, 2024	Navigated endoscopic resection of Eustachian tube mass
October 23, 2024	Discharge
April 2025	6-month follow-up: symptom resolution, no recurrence

On admission in September 2024, bilateral external auditory canals were unobstructed; the right tympanic membrane was retracted but intact. Bilateral tympanometry demonstrated Type A patterns, consistent with mild partial Eustachian tube obstruction without complete middle ear collapse. Pure tone audiometry showed mild-to-moderate sensorineural hearing loss at 1–3 kHz in the right ear and mild sensorineural hearing loss at 3–7 kHz in the left ear (Figure 1). This sensorineural component was considered pre-existing, while the conductive component related to Eustachian tube dysfunction was masked. Nasal endoscopy revealed a smooth, protuberant mass completely obstructing the right Eustachian tube orifice (Figure 2).

**Figure 1:**
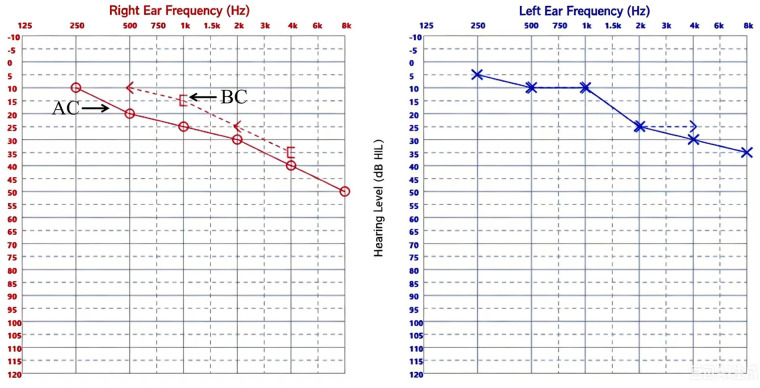
Pure tone audiogram. English abbreviations: AC, air conduction; BC, bone conduction. Mild-to-moderate sensorineural hearing loss in the right ear and mild hearing loss in the left ear.Standard audiometric symbols are adopted: open circle (○) for right ear air conduction, left angle bracket (<) for right ear bone conduction; cross (×) for left ear air conduction, right angle bracket (>) for left ear bone conduction. The horizontal axis represents sound frequency, and the vertical axis indicates hearing threshold in decibels.

**Figure 2:**
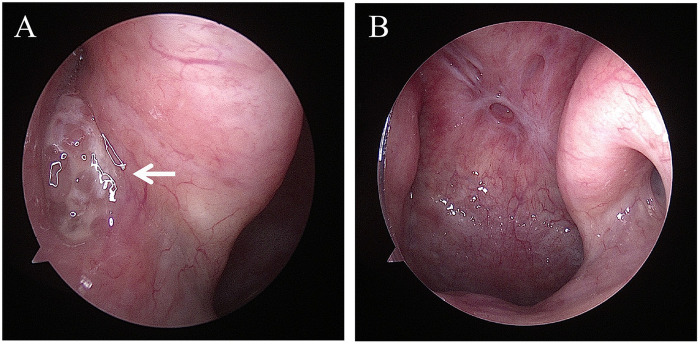
Nasal endoscopic image. **(A)** Right nasal cavity: a relatively smooth, protuberant mass completely obstructing the right Eustachian tube orifice. **(B)** Left nasal cavity: normal nasopharyngeal mucosa with a patent Eustachian tube orifice. Arrow indicates the obstructing mass at the right Eustachian tube orifice.

Nasopharyngeal MRI (late September 2024) demonstrated hypointense on T1-weighted and hyperintense on T2-weighted signals along the right Eustachian tube and torus tubarius. Post-contrast imaging showed an ill-defined, inhomogeneously enhancing nodule measuring 1.7 cm × 1.0 cm at the Eustachian tube orifice. Bilateral middle ear and mastoid effusion were noted, more prominent on the right; no cervical lymphadenopathy was detected (Figure 3).

**Figure 3:**
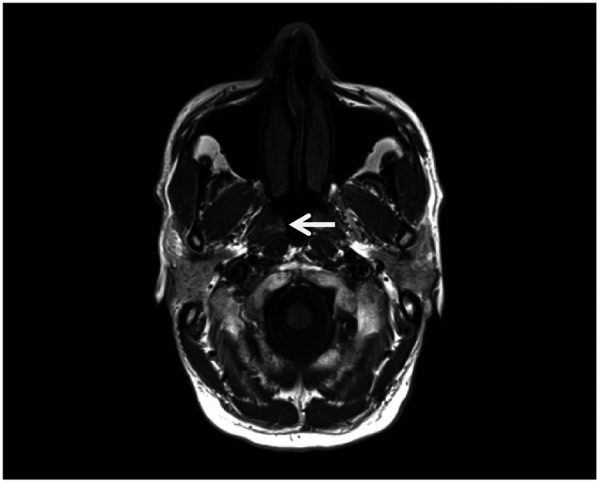
Nasopharyngeal MRI. Axial T1-weighted post-contrast; arrow indicates enhancing nodule at Eustachian tube orifice. An inhomogeneously enhancing nodule at the right eustachian tube.

After informed consent, navigated endoscopic resection of the right Eustachian tube mass was performed under general anesthesia in October 21, 2024, combined with septoplasty and inferior turbinate radiofrequency coblation for surgical exposure. Intraoperatively, a 1.0 cm × 1.4 cm soft mass was identified at the right Eustachian tube orifice, extending along the cartilaginous Eustachian tube. Frozen section confirmed benign lymphoid hyperplasia. The mass was completely resected under navigation guidance, and hemostasis was achieved. The postoperative course was uneventful.

Histopathological examination confirmed chronic mucosal inflammation with RLH, without malignant cells (Figure 4). Immunohistochemistry was expanded to exclude lymphoma and IgG4-related disease: CD20 (focal+), CD3 (focal+), CD4 (focal+), CD5 (focal+), CD10 (–), Bcl-6 (–), CD56 (–), CyclinD1 (–), Ki-67 (germinal center+), CD30 (–), CD138 (scattered+), IgG (scattered+), IgG4 (rare+), Pan-CK (epithelial+).

**Figure 4:**
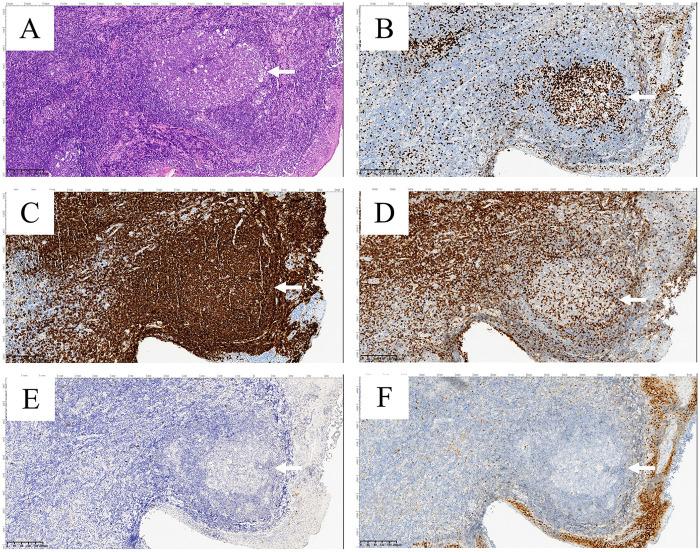
Histopathological and immunohistochemical findings of the lesion. **(A)** Hematoxylin and eosin (H&E) staining shows a reactive lymphoid follicle (arrow) with preserved architecture. **(B–F)** Immunohistochemical staining: **(B)** Ki-67 proliferative index highlights a high proliferation rate confined to the germinal center (arrow); **(C)** CD20 staining demonstrates diffuse positivity of B lymphocytes within the follicle (arrow); **(D)** CD3 staining shows scattered positive T cells in the interfollicular areas (arrow); **(E)** CD56 and **(F)** Cyclin D1 are negative in the lymphoid cells (arrows), supporting a reactive rather than neoplastic process.

The Eustachian Tube Dysfunction Questionnaire(ETDQ-7) (8) score improved from 32 preoperatively to 5 at 6-month follow-up, with complete resolution of aural fullness and tinnitus. Endoscopic follow-up at 6 months confirmed no recurrence; postoperative MRI was not performed given full symptom remission and unremarkable endoscopy, yet follow-up MRI will be scheduled upon new clinical complaints.

## Discussion

Lesions Lesions at the Eustachian tube orifice are mostly malignant, while benign lymphoproliferative lesions in this site are rare in clinical practice. These benign lesions often present with nonspecific symptoms and imaging features similar to malignant tumors, which can easily lead to misdiagnosis and unnecessary examination or treatment (9). This case describes a patient with benign lymphoid hyperplasia at the Eustachian tube orifice, focusing on clinical features, diagnostic difficulties, differential diagnosis, surgical management, and follow-up, to provide practical reference for similar clinical scenarios.

## Clinical features and diagnostic challenges

Patients with space-occupying lesions adjacent to the Eustachian tube orifice commonly present with nonspecific symptoms of Eustachian tube dysfunction—including aural fullness, conductive hearing loss, and recurrent or persistent otitis media. Due to their overlap with common otologic conditions, these manifestations are frequently misattributed to uncomplicated otitis media with effusion or primary Eustachian tube dysfunction during initial evaluation (10). In this case, the patient experienced progressive aural fullness and unilateral conductive hearing loss for five months. He was initially diagnosed with otitis media with effusion at a local hospital and received standard symptomatic management; however, his symptoms remained refractory. Subsequent high-resolution imaging and endoscopic evaluation revealed a well-circumscribed, enhancing mass obstructing the Eustachian tube orifice-later confirmed histopathologically as RLH.

Imaging examination is an important auxiliary method for evaluating Eustachian tube orifice masses (11). Malignant lesions such as nasopharyngeal carcinoma often show irregular borders, inhomogeneous enhancement, and possible invasion of surrounding tissues on MRI. Benign lymphoid hyperplasia, although less aggressive, can also present with ill-defined borders and inhomogeneous enhancement, which are similar to malignant tumors (12). These imaging characteristics often lead clinicians to suspect malignant tumors, resulting in repeated biopsies, enhanced imaging, and increased patient anxiety and medical burden. In recent years, clinical observations have suggested that benign lymphoid hyperplasia in the head and neck region is often associated with chronic inflammatory stimulation, and its imaging manifestations are easily confused with low-grade malignant tumors, requiring pathological examination for confirmation.

Another diagnostic challenge in this case is the inconsistency between tympanometry results and clinical manifestations. The patient had clinical manifestations of Eustachian tube obstruction and otitis media with effusion, but bilateral tympanometry showed Type A patterns. In general, patients with significant middle ear effusion and Eustachian tube obstruction often present with Type B or C tympanometry (13, 14). This case indicates that localized obstruction at the Eustachian tube orifice without complete middle ear collapse may lead to atypical tympanometry results, which can interfere with clinical judgment. Clinicians should combine multiple examination results, including endoscopy and imaging, for comprehensive evaluation.

## Differential diagnosis

Differential diagnosis is critical for avoiding misdiagnosis of Eustachian tube orifice masses (15–17). The main differential diagnoses for this case include:

- Nasopharyngeal carcinoma: The most common malignant tumor in the nasopharyngeal region, often accompanied by cervical lymphadenopathy, Epstein-Barr virus infection, and invasive growth on imaging.
- Extranodal NK/T-cell lymphoma: A rare aggressive lymphoma, often with necrotic lesions, destructive changes, and rapid progression.
- Adenoid/lymphoid hyperplasia: Usually diffuse hyperplasia of lymphoid tissue, common in children and adolescents, and rarely forms localized masses in adults.
- Benign nasopharyngeal polyps: Smooth, pedunculated lesions, usually non-enhancing on imaging, and easy to distinguish from solid masses.
- Inflammatory granuloma: Related to chronic rhinosinusitis or local inflammation, often with a history of recurrent inflammation.
- IgG4-related disease: A systemic fibroinflammatory disease, which can involve the head and neck region, often with elevated serum IgG4 and characteristic pathological changes.
- Tornwaldt cyst: A congenital midline cyst in the nasopharynx, usually cystic and non-enhancing on imaging.

For patients with Eustachian tube orifice masses such as in this individual case, detailed medical history collection, comprehensive physical examination, and targeted auxiliary examinations are necessary. Pathological examination, including biopsy and immunohistochemistry, is the gold standard for distinguishing benign from malignant lesions (18).Recent clinical experience emphasizes that for suspicious benign lymphoproliferative lesions, repeated biopsy or intraoperative frozen section examination can help confirm the diagnosis and reduce the risk of misdiagnosis (19).

## Surgical management and postoperative follow-up

For localized benign masses at the Eustachian tube orifice, surgical resection is both diagnostic and therapeutic (20). Endoscopic surgery has become the mainstream method for treating nasopharyngeal and Eustachian tube lesions due to its advantages of minimally invasive, clear surgical field, and rapid postoperative recovery (21). In this case, navigation-assisted endoscopic resection was performed, which helped accurately locate the mass, ensure complete resection, and reduce damage to surrounding normal tissues. The postoperative course was smooth, and the patient's symptoms were significantly relieved (22).

Postoperative follow-up is a critical component for evaluating surgical efficacy and detecting potential recurrence. For benign lesions such as RLH, multiple case series have demonstrated excellent outcomes after complete surgical excision. In a case series of 105 patients with benign sinonasal tumors followed for a mean of 41 months, endoscopic endonasal surgery achieved a low overall recurrence rate of 7.6%, with the majority of patients remaining disease-free throughout the follow-up period (23). More specifically, for RLH lesions, a case series of 5 patients with benign RLH of the caruncle followed for a mean of 48 months reported no local recurrence in any patient (24). Another series of 3 pediatric patients with ocular adnexal RLH followed for 9–15 months also demonstrated no evidence of recurrence, transformation, or systemic involvement (25). Based on these data, we estimate the 5-year recurrence risk of completely excised benign RLH to be less than 10%, with most reported cases showing 0% recurrence in mid-term follow-up.

Follow-up strategy (institutional standpoint): Given the rarity of RLH localized to the Eustachian tube orifice and the lack of high-level evidence, we propose the following institutional protocol for postoperative surveillance, which combines clinical assessment, endoscopic evaluation, and symptom-guided imaging:

- Clinical tools: The ETDQ-7 questionnaire should be administered at each follow-up visit to monitor symptom control. Nasal endoscopy serves as the primary imaging modality for direct visualization of the nasopharynx. Routine MRI is not required for asymptomatic patients with negative endoscopic findings; instead, MRI should be reserved for patients with new or progressive symptoms (e.g., recurrent aural fullness, hearing loss, or tinnitus) or suspicious endoscopic findings.
- Timeline: We recommend the first follow-up evaluation at 3–6 months postoperatively, combining endoscopic examination and ETDQ-7 assessment. Subsequently, endoscopic monitoring should be performed every 6–12 months for the first 2–3 years, given that most recurrences of benign nasopharyngeal lesions occur within this window. For patients who remain symptom-free with negative endoscopy beyond 3 years, surveillance can be transitioned to annual endoscopic checks or converted to symptom-triggered follow-up.

Importantly, because the literature on Eustachian tube orifice RLH is limited to isolated case reports, these recommendations represent our institutional view based on available evidence from analogous benign lymphoproliferative lesions and our own clinical experience. Individualized adjustments should be made according to each patient's clinical course and symptoms.

## Strengths and limitations

Due to the extreme rarity of RLH localized exclusively at the Eustachian tube orifice, large prospective clinical cohorts are unavailable, which hinders the formulation of unified diagnostic criteria and standardized therapeutic guidelines based on high-volume clinical data. Under such circumstances, well-documented individual case reports play an irreplaceable role in accumulating clinical evidence and filling the existing knowledge gap of this uncommon disease. As a single-case report, our work cannot establish definitive treatment norms, but detailed documentation of clinical manifestations, imaging features, pathological findings and surgical outcomes helps enrich available clinical data for this rare lesion. The 6-month follow-up in this case is relatively short. However, based on analogous data from benign RLH at other sites, the expected recurrence risk after complete excision is low (estimated <10% at 5 years) (23, 24). Long-term recurrence risk nevertheless requires further case accumulation for conclusive evaluation, and we have therefore proposed an institutional follow-up protocol that includes endoscopic surveillance every 6–12 months for 2–3 years, followed by symptom-triggered monitoring thereafter.

## Conclusion

Benign RLH at the Eustachian tube orifice is a rare clinical entity that mimics malignant tumors in clinical manifestations and imaging features. Clinicians should maintain a high degree of vigilance for such rare lesions and rely on pathological examination for definitive diagnosis.

Endoscopic surgical resection is effective for relieving symptoms and confirming diagnosis. The postoperative prognosis is favorable after complete resection, with a low risk of recurrence. Although long-term regular follow-up is recommended to monitor for potential late recurrence, the intensity of surveillance should be balanced against the benign nature of the lesion. Timely and accurate diagnosis helps avoid misdiagnosis and overtreatment, thereby optimizing patient outcomes.

## Data Availability

The original contributions presented in the study are included in the article/Supplementary Material, further inquiries can be directed to the corresponding author.
